# Life course socio-economic position and quality of life in adulthood: a systematic review of life course models

**DOI:** 10.1186/1471-2458-12-628

**Published:** 2012-08-09

**Authors:** Claire L Niedzwiedz, Srinivasa V Katikireddi, Jill P Pell, Richard Mitchell

**Affiliations:** 1 Institute of Health and Wellbeing, College of Medical, Veterinary and Life Sciences, University of Glasgow, Glasgow, UK; 2 Medical Research Council/Chief Scientist Office Social and Public Health Sciences Unit, Glasgow, UK

## Abstract

**Background:**

A relationship between current socio-economic position and subjective quality of life has been demonstrated, using wellbeing, life and needs satisfaction approaches. Less is known regarding the influence of different life course socio-economic trajectories on later quality of life. Several conceptual models have been proposed to help explain potential life course effects on health, including accumulation, latent, pathway and social mobility models. This systematic review aimed to assess whether evidence supported an overall relationship between life course socio-economic position and quality of life during adulthood and if so, whether there was support for one or more life course models.

**Methods:**

A review protocol was developed detailing explicit inclusion and exclusion criteria, search terms, data extraction items and quality appraisal procedures. Literature searches were performed in 12 electronic databases during January 2012 and the references and citations of included articles were checked for additional relevant articles. Narrative synthesis was used to analyze extracted data and studies were categorized based on the life course model analyzed.

**Results:**

Twelve studies met the eligibility criteria and used data from 10 datasets and five countries. Study quality varied and heterogeneity between studies was high. Seven studies assessed social mobility models, five assessed the latent model, two assessed the pathway model and three tested the accumulation model. Evidence indicated an overall relationship, but mixed results were found for each life course model. Some evidence was found to support the latent model among women, but not men. Social mobility models were supported in some studies, but overall evidence suggested little to no effect. Few studies addressed accumulation and pathway effects and study heterogeneity limited synthesis.

**Conclusions:**

To improve potential for synthesis in this area, future research should aim to increase study comparability. Recommendations include testing all life course models within individual studies and the use of multiple measures of socio-economic position and quality of life. Comparable cross-national data would be beneficial to enable investigation of between-country differences.

## Background

A life course approach to health recognizes the importance of early and later life exposures in identifying risk and protective processes operating throughout an individual’s lifetime [1]. Exposure to low socio-economic position over the life course has been shown to influence a range of health outcomes, including cause-specific mortality and cardiovascular disease [2,3]. For the purpose of this review, socio-economic position (SEP) refers to the socially derived economic factors that influence the positions individuals hold within a stratified society, measured by individual or household level indicators such as education level and occupation [4]. In chronic disease epidemiology, several conceptual models have been developed to help elucidate the mechanisms underlying life course socio-economic effects on health. These provide a foundation for investigating life course effects, however their effects are difficult to differentiate as they are empirically interlinked [5].

The *accumulation* model hypothesizes that early and later adverse socio-economic experiences have a cumulative, dose–response effect on later outcomes [6]. The *latent* model (or critical period) suggests that adverse socio-economic circumstances during childhood have an independent, detrimental effect on health, over and above current circumstances [7]. *Pathway* models emphasize the importance of trajectories across the life course and are proposed if the influence of childhood SEP is attenuated after taking into account later conditions. *Social mobility* models are usually divided into *intra-generational* and *inter-generational*. Inter-generational mobility refers to a change in social class between generations, often measured by comparing parental social class to own social class in adulthood. Intra-generational mobility is the movement between different social classes in adulthood, such as the first and last occupation. No consensus regarding the health consequences of social mobility exists. It has been proposed that downward mobility may negatively impact on mental health and wellbeing [8], whereas others suggest that any movement between social classes will result in increased mental strain and illness [9]. Another hypothesis states that mobility itself does not have an independent influence, but mobile individuals eventually experience levels of health and wellbeing between those of their current class and class of origin, closest to the current social class [10,11].

A positive association between current SEP and quality of life has been demonstrated using subjective wellbeing, life and needs satisfaction approaches [12-17]. Subjective wellbeing is defined as the balance between positive and negative affect [18]. Life satisfaction is the cognitive evaluation of one’s life, which involves the comparison between one’s aspirations and achievements [19]. Needs satisfaction approaches derive from Maslow’s theory of human need, which proposes that once basic human needs are satisfied, such as food and safety, humans strive for higher needs such as self-happiness and esteem [19,20]. Most quality of life research has focused on contemporary influences and less is known regarding the effect of different life course socio-economic trajectories on later quality of life. This is of growing interest to academics and policymakers, as subjective quality of life is now considered an important indicator for the evaluation of interventions across several disciplines, and governments are increasingly adopting the measurement of national subjective wellbeing to inform policy decisions [21,22]. However, there is no consensus regarding the optimal measure of quality of life and it remains an ill-defined concept [23].

Broadly, subjective quality of life involves the self-evaluation (expression of satisfaction or discontent, values and perceptions) of one’s personal circumstances in life [19]. Subjective wellbeing (positive and negative affect), happiness, life and needs satisfaction measures are often used as key indicators of subjective quality of life. Numerous tools have been designed to capture these, such as the Satisfaction with Life Scale [24], Ryff’s psychological wellbeing scale [25] and CASP-19, a needs satisfaction measure comprised of control, autonomy, self-realization and pleasure domains [23].

This systematic review aimed to assess whether evidence supported an overall relationship between life course socio-economic position and quality of life during adulthood and if so, whether there was support for one or more life course models.

## Methods

PRISMA guidelines for the reporting of systematic reviews were followed [26] and a review protocol was developed and updated as necessary throughout the review process and is available from the authors on request.

### Search strategy

Articles were identified by searching (via Ovid) the electronic databases Medline (1948-present), Embase (1947-present) and PsycInfo (1987-present). Additional searches were executed in Web of Science and Cambridge Scientific Abstracts (CSA) Illumina. Web of Science covered the databases Science Citation Index Expanded (1945-present), Social Sciences Citation Index (1956-present), Arts & Humanities Citation Index (1975-present), Conference Proceedings Citation Index- Science (1990-present) and Conference Proceedings Citation Index- Social Science & Humanities (1990-present). CSA Illumina covered Applied Social Sciences Index and Abstracts (1987-present), International Bibliography of the Social Sciences (1951-present), CSA Sociological Abstracts (1952-present), and Worldwide Political Science Abstracts (1975-present). All searches were carried out on January 2^nd^ 2012 and limited to English language articles. No restrictions were placed on the publication date of articles. Reference lists of included articles were checked for any additional articles and citations were accessed via Google Scholar and checked manually.

Searches included terms used to describe SEP, such as ‘social class’ and ‘occupation’, combined with terms used to describe the life course and quality of life. Relevant MeSH headings were used when available. See Additional file 1 for a full example of the search strategy executed in Medline.

### Eligibility criteria

Studies were included if they met the following criteria: primary studies published in a scholarly journal; based on populations within industrialized countries as defined by Organization for Economic Co-operation and Development criteria [27]; reported subjective quality of life as an outcome (using indicators separate from physical health such as wellbeing, life satisfaction or specific quality of life scales such as CASP-19); reported outcomes in males and/or females aged 25 years or over (as this represented an adult population in which individuals were likely to have completed their education); contained individual or household measures of SEP from at least two time points (childhood and adulthood, or two time points in adulthood, regardless of the length of time between measurement points or whether they specified a particular life course model).

Articles were excluded if they contained only qualitative data, were review articles, did not specify any information regarding the age of participants, included only area-level or subjective measures of social status (as these were considered different constructs [28]), only looked at employment status, job or income mobility (without a measure of social class), contained only measures of physical health-related quality of life or which did not separate between physical and mental components of health-related quality of life (as we were interested in outcomes capturing aspects of quality of life separate from physical health), included only individuals with specific health conditions (e.g. dementia, psychiatric illness) as their population of interest, or contained only outcomes relating to psychiatric symptoms (e.g. psychological distress or depressive symptoms).

### Study selection and data extraction

Title and abstract screening for immediately irrelevant articles was performed by one reviewer (CLN). Two reviewers (CLN and SVK) independently assessed the full-texts of articles short-listed against the eligibility criteria. Disagreements were resolved by consensus. All records were stored in Endnote X4. An Excel proforma was developed to assist in the data extraction procedure and included: the publication information (authors, year, journal), study characteristics (sample size, study design, response and attrition rates, time period), participant demographics (age at recruitment, gender, country), measurement of SEP (collection method, age at measurement, missing data), outcomes (summary measure such as mean quality of life scores or odds ratio of experiencing low quality of life, collection method, age at measurement, missing data), analysis methods (statistical techniques, variables controlled for, treatment of missing data) and results. Data were extracted by CLN and checked by SVK.

### Quality appraisal

Quality appraisal was performed using an adapted version of the ‘Quality Assessment Tool for Quantitative Studies’ [29]. The following items were used to assess the quality and risk of bias within studies: sampling method, sample representativeness, study design, response rates, attrition rates and reasons (including death and loss to follow-up), whether the characteristics of those lost to attrition or non-response differed from those of responders, measurement of SEP and quality of life variables, reporting of missing data, and variables controlled for in the analysis to reduce confounding. Given the limitations associated with scoring criteria [30], the quality of articles was initially considered during the synthesis process. Three key items, which we considered the most important quality criteria, were then selected to provide an overall indication of study quality using a rating system. These were: the response and attrition rates, measurement of SEP, and sample size. A grade of higher, average or lower quality was given to studies based on the sum of scores for these items (see Additional file 2 for full details of the rating system). The quality appraisal of studies did not differ between the two methods of assessment (synthesis based on all quality items extracted and the rating system using the three key items).

### Data analysis and presentation

Studies differed in terms of the measures of SEP, outcomes, time points considered and analysis techniques implemented. For these reasons, meta-analysis was not appropriate and narrative synthesis was used. Studies were categorized based on the life course model analyzed: accumulation, latent, pathway, or social mobility (inter-generational and/or intra-generational). Studies were grouped into the relevant life course model based on their aim, analytic approach and findings, similar to the method by Pollitt et al. [3]. We also compared our own classifications with those of the authors, if provided, but no conflicting groupings occurred. If more than one model was assessed within the same study, the results are presented under multiple categories according to the life course models investigated. It should be noted that positive results may be found for more than one model within the same study. A summary of the evidence is presented under each life course model (studies considered higher quality are described first), followed by a discussion of the key issues relating to the comparison of results.

## Results

### Search results

**Figure 1:**
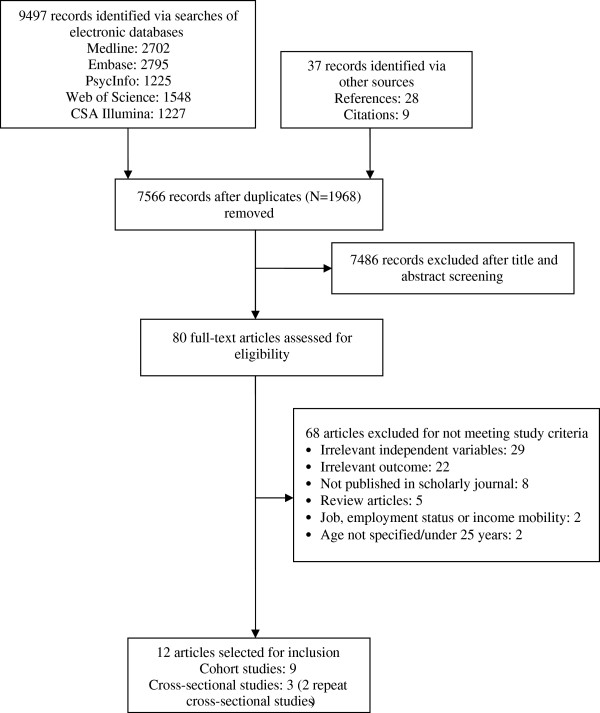
Flow diagram of article identification, screening, eligibility and inclusion.

The electronic searches identified 7,529 publications, following removal of duplicates (Figure 1). Thirty-seven additional records were identified via reference and citation checks. Following title and abstract screening, 7,486 irrelevant records were excluded. Examples of records excluded at this stage included those assessing health-related quality of life in disease-specific patient groups. Eighty full-text articles were assessed for eligibility, of which, 12 were selected for inclusion. Eleven articles were included by CLN; an additional article was identified by SVK and included following discussion. See Additional file 3 for the list of excluded full-text articles and the corresponding reasons for exclusion.

### Study characteristics

The 12 articles used 10 different datasets and represented findings from a total of 35,022 individuals (Table 1). See Additional file 4 for further details of each study’s variables, analysis techniques and results. The findings span five countries: the United States, United Kingdom, Finland, Sweden and Spain. Two studies used data from the same repeat cross-sectional surveys of the Helsinki Health Study, but used different SEP measures and analysis techniques [31,32]. Two studies were also published using the Swedish Individual Development and Adaptation Cohort [33,34]; Johansson et al. (2007) included a later survey wave and different outcome. The publications by Breeze et al. (2001) and Singh-Manoux et al. (2004) were based on the Whitehall I and II studies respectively, which included only British civil servants as the target population [6,35]. In the Whitehall II study there is a 10-fold difference in salary between those at the top and bottom of the civil service hierarchy [36]. Several outcomes were used including wellbeing (using self-esteem, self-acceptance or positive psychological functioning indicators), life satisfaction, CASP-19 and the mental component summary (MCS) of SF-36. Nine studies assessed a single life course model and three assessed more than one.

**Table 1:** summary of articles included in the systematic review categorized by life course model

**First author, year, reference Country**	**Study design N**	**Quality Rating**	**Gender**	**Measures of SEP**	**Model**	**Outcomes**	**Results**
Mäkinen 2006 [32] Finland	Repeat cross-sectional N = 8970	Average	20% male	Childhood SEP: parent’s education level & childhood circumstances. Adulthood SEP: own education level	A	SF-36 MCS	No support.
Otero-Rodríguez 2010 [40] Spain	Cohort N = 2117	Average	45% male	Childhood SEP: father’s occupation. Own education level. Adulthood SEP: current/last occupation of household head	A	Change in SF-36 MCS	Support for accumulation model – risk of decline in MCS increased linearly with increasing number of low SEPs.
Singh-Manoux 2004 [6] United Kingdom	Cohort N = 6128	Average	72% male	Childhood SEP: father’s occupation & childhood socioeconomic circumstances. Own education level. Adulthood SEP: employment grade	A	SF-36 MCS	Support for accumulation model among men only – risk of being in lowest quintile increased linearly with increasing number of low SEPs.
Huurre 2003 [41] Finland	Cohort N = 1592	Higher	45% male	Childhood SEP: father's occupation. Adulthood SEP: own occupation	L	Wellbeing	Support for latent model among women only – lower childhood SEP associated with poorer wellbeing.
Marmot 1998 [38] United States	Cross-sectional N = 3032	Average	48% male	Childhood SEP: parent’s education level. Adulthood SEP: own education level	L	Wellbeing	Some support for latent model among women who had mothers with lowest education – lower childhood SEP associated with poorer wellbeing.
Otero-Rodríguez 2010 [40] Spain	Cohort N = 2117	Average	45% male	Childhood SEP: father’s occupation. Own education level. Adulthood SEP: current/last occupation of household head	L	Change in SF-36 MCS	Support for latent model – low childhood SEP associated with highest risk of decline and improvement in MCS.
Laaksonen 2007 [31] Finland	Repeat cross-sectional N = 8970	Average	20% male	Childhood SEP: parent’s education level. Adulthood SEP: own education level, income & occupation	L & P	SF-36 MCS	No evidence for latent model in men or women. Support for pathway model in men & women – higher adulthood SEP associated with increased risk of low MCS.
Mäkinen 2006 [32] Finland	Repeat cross-sectional N = 8970	Average	20% male	Childhood SEP: parent’s education level. Adulthood SEP: own education level	L & P	SF-36 MCS	Support for latent model in women only – higher childhood SEP associated with increased risk of low MCS. No support for pathway model in men or women.
Blane 2004 [39] United Kingdom	Cohort N = 254	Poorer	47% male	Inter-generational mobility: father’s occupation & respondent’s longest held occupation. Intra-generational mobility: respondent’s occupation aged 25 & 50 years	SM (inter & intra)	CASP-19	No support.
Otero-Rodríguez 2010 [40] Spain	Cohort N = 2117	Average	45% male	Inter-generational mobility: father’s occupation & current or last occupation of household head	SM (inter)	Change in SF-36 MCS	Support for social mobility – upwardly mobile more likely to experience change in MCS scores. No evidence for downwardly mobile.
Runyan 1980 [37] United States	Cohort N = 91	Poorer	49% male	Inter-generational mobility: father’s occupation & respondent’s occupation aged around 38 years	SM (inter)	Life satisfaction	No support.
Breeze 2001 [35] United Kingdom	Cohort N = 7041	Average	100% male	Intra-generational mobility: employment grade at baseline & employment grade at retirement	SM (intra)	SF-36 MCS	Support for intra-generational effect – upwardly mobile less likely to have poor MCS score.
Houle 2011 [42] United States	Cohort N = 4992	Higher	100% male	Intra-generational mobility: occupation aged around 36 years & 52 years	SM (intra)	Wellbeing	No support intra-generational effect – mobile individuals more likely to report wellbeing resembling current class than prior class.
Huang and Sverke 2007 [33] Sweden	Cohort N = 291	Average	100% female	Intra-generational mobility: respondent’s occupational history from ages 16 to 43 years	SM (intra)	Life satisfaction	No support.
Johansson 2007 [34] Sweden	Cohort N = 514	Average	100% female	Intra-generational mobility: respondent’s occupational history from ages 16 to 43	SM (intra)	Life satisfaction & wellbeing	Life satisfaction: no support. Wellbeing: some support – downwardly mobile reported lower wellbeing.

*A* = accumulation; Inter = inter-generational; *Intra* = Intra-generational; *L* = latent; *MCS* = mental component summary; *N* = Sample size; *P* = pathway; *SEP* = socio-economic position; *SF-36* = short-form 36; *SM* = social mobility.

### Quality assessment summary

The sample size of included studies ranged from 91 [37] to 8,970 individuals [31,32], with a median of 2,117 individuals. The attrition rate of studies ranged from 3% over 14 years [6], to 57% over 27 years [37]. Response rates for the last wave of study reported varied from 61% [38] to 90% [39], median 76.5%. See Additional file 5 and Additional file 6 for details of the full quality appraisal and ratings for each article.

### Accumulation model

Three studies tested the relationship between life course SEP and quality of life using the accumulation model [6,32,40]. All three studies were assessed as being of average quality. Singh-Manoux et al.(2004) found some support for the model among men; as the number of occasions in a low SEP increased, the more likely the respondent was to report MCS scores in the lowest category [6]. A significant linear trend was also identified. However, no evidence was found among women, with the highest odds ratio being found in those who had an intermediate SEP score. Highest odds ratios for men were evident in trajectories that began in an intermediate or low SEP during childhood and moved to a high SEP in adulthood, perhaps indicating support for latent or social mobility models. Otero-Rodriguez et al. (2011) used polytomous logistic regression models with risk of decline, no change (reference category) and improvement in MCS scores over a two year period as the outcome. They found the risk of decline was highest in individuals reporting three low SEPs at key points in the life course, compared to those with one low SEP, and a significant linear trend was identified [33]. The risk of improvement was also highest in those reporting three low SEPs, but no linear trend was identified. Mäkinen et al. (2006) found no support for the accumulation model using education level as the only indicator of SEP [32]. The prevalence of MCS scores with ‘limited functioning’ was slightly increased for men and women who experienced a low childhood SEP and high adulthood SEP, compared to those with a low SEP at both time points.

In summary, mixed evidence was found for the accumulation model and differing results were found between genders. The contradictory results of Mäkinen et al. (2006) could be explained by heterogeneity between studies in terms of the SEP measures and study contexts. Mäkinen et al. (2006) used the respondent’s own education level and parental education level, whereas Singh-Manoux et al. (2004) used a combination of the respondent’s employment grade, education level, and parental occupation. Perhaps education levels alone do not capture accumulated socio-economic disadvantage to the same extent as taking parental and respondent’s occupations into account. Education level may also be considered a more distal indicator of SEP, which reflects both prior cognitive ability and parental SEP. Cohort differences in the meaning of education between generations may also exist. For example, Mäkinen et al. (2006) used a Finnish population aged 40 to 60 years old. It is likely that the respondent’s parents completed their education at a time before the rapid expansion in higher education. Over half of respondents had high education levels, but over half of their parents had low education levels [32]. Therefore, having low education level in the parental generation may not confer disadvantage in the same way as in the respondent’s generation, where a higher education level may confer higher social status. The context of the paper by Singh-Manoux et al. (2004) is also important to consider. They used the Whitehall II study based on British civil servants, in which the social hierarchy is more amenable to measurement due to the distinct civil service grades.

### Latent and pathway models

Five studies assessed life course effects on quality of life using the latent model [31,32,38,40,41]. Laaksonen et al. (2007) and Mäkinen et al. (2006) used the same dataset, but different methods, to simultaneously test the pathway model [31,32]. One study was assessed as being of higher quality [41] and four were of average quality [31,32,38,40].

Huurre et al. (2003) found poorer mean wellbeing scores among female respondents reporting their parents had a manual occupation, compared to non-manual occupation [41]. When adjusting for the respondent’s own current social class, this difference remained significant. The effect was not observed among men. Otero-Rodriguez et al. (2011) found that individuals reporting a low childhood SEP were more likely to experience change in MCS scores compared to those with a high childhood SEP [40]. Low education was also independently associated with a decline in MCS scores, but not improvement. Adulthood SEP did not have an independent effect on change in MCS scores. Marmot et al. (1998) found no support for the latent model among men and some support among women [38]. Using father’s education, the odds ratios for low wellbeing among women were not significantly different between education levels. However, using mother’s education level, the odds ratio for low wellbeing was significantly elevated among women with the lowest educated mothers. This indicates that, for women, a latent effect of low childhood SEP may operate via the mother’s SEP.

Conflicting results were found between the two studies by Laaksonen et al. (2007) and Mäkinen et al. (2006) [25,26]. Mäkinen et al. (2006) used a similar method to the above studies to test the latent model and identified support among women, but in the opposite direction to that expected [32]. Women who had a low or intermediate SEP in childhood exhibited lower odds of having MCS scores in the ‘low functioning’ range, compared to those recording a high childhood SEP. This effect was not present in men. No significant pathway effect was suggested in men or women. On the other hand, Laaksonen et al. (2007) implemented a structural equation modeling approach and found no evidence for a direct effect of childhood SEP on MCS means [31]. However, they identified a direct effect of adulthood SEP in the opposite direction hypothesized; higher adulthood SEP was associated with poorer MCS scores in both men and women. The results indicated evidence for a pathway effect in which childhood SEP influenced MCS scores, via adulthood SEP which acted as a mediator. However, the authors highlighted that their method of analysis did not permit the calculation of confidence intervals around the indirect effects estimates and cautioned that this result should be interpreted with care.

To summarize, mixed evidence was found for a latent effect, with an indication that the model may be more plausible among women. Care should be taken when making overall conclusions due to differing exposure and outcome variables and inconsistent analysis methods. The results by Mäkinen et al. (2006) and Laaksonen et al. (2007) highlight that contrasting results may be found using different analysis techniques and different measures of SEP, despite using the same data. Modeling SEP as a latent variable including education, occupation and income, as in the paper by Laaksonen et al., may provide a more accurate indication of adulthood SEP and act to diminish any effect of childhood SEP. However, this does not explain why Mäkinen et al. (2006) found an association in the opposite direction to that expected. The two studies used data from a Finnish public sector occupational cohort where 80% of participants were female. Compared to the studies by Huurre et al. (2003) and Marmot et al. (1998), the women employed may work in jobs requiring higher demand, with higher status and stress. This could explain why Laaksonen et al. (2007) identified evidence for a pathway effect in which current, rather than childhood socio-economic circumstances had most influence on quality of life.

### Social mobility models

Seven publications investigated the effect of social mobility on quality of life. Inter-generational mobility was tested in three [37,39,40], intra-generational mobility in five [33-35,39,42] and one assessed both types [39]. The quality of studies varied considerably. First, the results for inter-generational mobility will be presented, followed by those for intra-generational mobility.

#### Inter-generational mobility

Few studies investigated the effect of inter-generational mobility. Otero-Rodriguez et al. (2011) found that the upwardly mobile had highest odds of experiencing a change MCS scores, but no evidence for an effect of downward mobility was identified [40]. Blane et al. (2004) found no support for an effect of inter-generational mobility on mean CASP-19 scores [39]. The scores differed very little between those who were upwardly and downwardly mobile, or who had the same SEP at both time points. However, the number of individuals in some mobility categories was small. The study was rated as being poorer quality due to its low sample size and poor response rate in the second wave. Runyan (1980) also found no supportive evidence for an effect of mobility on mean life satisfaction [37]. The outcome was measured using retrospective recall of life satisfaction levels from the previous four years, which may have introduced recall bias in the outcome. Low numbers were also apparent in most mobility categories. The study was ranked as poorer quality, exemplified by a low mark in all quality criteria items.

#### Intra-generational mobility

No supportive evidence was found in three of five studies assessing intra-generational mobility effects on quality of life [33,39,42]. One study was considered higher quality [42], three average quality [33-35] and one poorer quality [39]. Neither upward nor downward mobility was associated with wellbeing in the study by Houle [42]. However, socially mobile individuals were twice as likely to report levels of wellbeing that resembled non-mobile individuals in their social class of entry (or current class), rather than their prior class. Further, when controlling for the number of years in the current social class, the effect became stronger. Breeze et al. (2001) found that the upwardly mobile were less likely to report poor MCS scores [35]. The effect size among those who were in a low grade at baseline and moved to a higher grade at retirement was smaller, compared to those from a middle grade who moved to a higher grade. Huang and Sverke (2007) found no difference in mean life satisfaction between those who had upward, downward and stable mobility patterns using two waves of a Swedish cohort study [33]. Johansson et al. (2007) also found no difference in life satisfaction outcomes using three waves of the same study, but the upwardly mobile reported significantly higher mean wellbeing scores compared to those who were downwardly mobile [34]. The study by Johansson et al. (2007) was slightly strengthened by a larger sample size compared to Huang and Sverke (2007). Blane et al. (2004) found no supportive evidence for an effect of intra-generational mobility on mean CASP-19 scores [39].

To summarize, mixed evidence was found for an effect of intra-generational mobility on quality of life. Only one study was mixed gender, but did not control for gender effects [39]. Johansson et al. (2007) investigated wellbeing and life satisfaction outcomes and found the results differed depending on the measure used [34]. Additionally, Breeze et al. (2001) included only male British Civil Servants as the target population, where upward mobility may have been more common [29]. Country-level factors may also have influenced the results. As the authors highlighted, the women included in the studies by Huang and Sverke (2007) and Johannsson et al. (2007) were relatively privileged compared to other countries, with greater choice regarding their career construction and benefitting from better parental leave and availability of childcare [33]. Therefore, upward mobility may have been easier for these women, compared to countries such as the United States and the United Kingdom.

## Discussion

This systematic review used life course models derived from chronic disease epidemiology to assess the relationship between life course socio-economic position and quality of life during adulthood. An overall relationship was suggested by the evidence, but results for each life course model were mixed. Supportive evidence was found for the latent model among women only, but results were contradictory. Some studies indicated that low childhood SEP was associated with poorer adult quality of life, but others found high childhood SEP to be linked with poorer outcomes. Social mobility models were generally not supported, but some studies investigating intra-generational mobility did identify an effect. There was a suggestion that upwardly mobile individuals experienced higher quality of life, compared to those who moved downward or remained in the same position. However, one higher quality study which modeled separate mobility effects found no effect of intra-generational mobility; mobile individuals were more likely to report quality of life levels closer to their current class, rather than their prior class. High quality studies addressing inter-generational mobility were lacking. Few studies addressed accumulation and pathway effects and heterogeneity of these studies resulted in limited synthesis.

A similar systematic review focusing on cardiovascular outcomes found consistent support for an accumulation effect of socio-economic adversity on cardiovascular disease risk and moderate support for a latent effect of low childhood SEP on increased cardiovascular disease risk factors, morbidity and mortality [3]. Little support was found for a unique influence of social mobility, although they did not distinguish between inter- and intra-generational effects. Our review particularly lacked studies investigating accumulation effects, thus additional research is required to fully assess this hypothesis for quality of life outcomes. Regarding social mobility effects, the study by Houle (2011) is consistent with the literature which demonstrates that mobile individuals tend to have health outcomes between their social class of origin and destination, so social mobility has a constraining effect on health inequalities [11,43-45]. Further research would be useful to investigate whether this applies to other quality of life outcomes and in other countries with differing levels of social mobility.

A particular strength of the systematic review was the number of databases searched. However, the grey literature was not explored and only quantitative English language articles were included. Important unpublished articles and foreign language studies may exist which were not considered. It is also possible that key insights into the individual experience of different life course socio-economic trajectories may be provided by qualitative studies. Quality assessment was performed by describing all quality items relevant to a study and by ranking studies based on key quality appraisal items. The latter system may be crude and opinions are likely to differ regarding the key criteria. However, compared to the pure description of studies, we feel the criteria enable the reader to better discern between higher and lower quality studies.

A number of limitations relating to quality of life research require highlighting. Due to the ambiguous nature of quality of life, it is possible that studies measuring outcomes such as life satisfaction and wellbeing are capturing different concepts, or various domains of the same concept [46]. It is therefore suggested that researchers try to include a variety quality of life measures in their studies, to investigate whether associations differ between indicators. Cohort studies which record quality of life at several time points throughout adulthood would also be an improvement, especially when investigating intra-generational mobility effects.

Cultural differences may also exist in the understanding of survey questions and the degree to which expressing satisfaction is believed desirable [24,47]. Populations from a range of countries were included in the review and the results between countries became difficult to interpret. A number of macro-level factors, such as welfare state arrangements and educational policies, may influence the degree to which life course socio-economic conditions shape later quality of life. To enable investigation of between-country differences, there is a need for the increased collection, harmonization and utilization of comparable cross-national data. Studies that take into account potential cultural differences in reporting styles, as well as local and national context are required [48].

Although methodological limitations in life course models have been noted [49], they provide a useful, albeit simplified, foundation to investigate potential life course effects. As previously suggested, it is recommended that all life course models are considered within individual studies, to prevent patterns of association being overlooked [3]. Separating the effects of different models is difficult as they are empirically interlinked; however this need not be the key objective and perhaps risks their reification. Examining the evidence for each model together can help to obtain a more complete understanding of any relationship, refine the concepts and generate new hypotheses [50]. Studies which include multiple measures of SEP are also recommended, as results may vary depending on the specific indicator used [3,51]. It is important that when including multiple measures, such as social class and education, the sociological meaning of these is considered and related to the specific hypotheses and causal pathways under study [52]. It may also be useful to consider the length of time spent in each social class to better quantify accumulation and mobility effects. In addition, future empirical work may benefit from considering the life course principles of biological and social plausibility [5]. Cross-sectional research often relies on the retrospective recall of socio-economic variables. However, studies have shown that this may not be a major issue especially when using methods to facilitate recall of events, such as the life-grid method, and may only lead to the under-estimation of associations [53-55]. Cohort studies which record socio-economic information from birth to old age would be ideal, although time-consuming and expensive. These are not without problems, however, often suffering from increasing attrition over time. This may lead to selection bias or a ‘healthy survivor effect’ where individuals with poorer outcomes are selected out of studies. However, better understanding of micro- and macro-level factors which nurture high quality of life in those exposed to adversity across the life course has key policy relevance.

## Conclusions

Evidence suggested an overall relationship between life course socio-economic position and quality of life during adulthood, but results for each life course model were mixed. Among women, evidence was found to support the latent model and social mobility models were generally not supported. There was a lack of studies testing accumulation and pathway effects. Future research should aim to assess all life course models within studies and use multiple measures of SEP and quality of life. Comparable cross-national data would be beneficial in reducing study heterogeneity between countries and provide insight into potential contextual influences on the relationship between life course socio-economic factors and later outcomes. Better understanding of life course influences has the potential to contribute to improved policy and interventions to address health inequalities.

## Competing interests

The authors declare that they have no competing interests

## Authors’ contributions

CLN, JPP and RM conceived the review and contributed to its methodology. CLN conducted the literature searches, selected and categorized studies, extracted and analyzed the data and wrote the manuscript. SVK acted as second reviewer and was involved in the short-listing of articles and checked extracted data. All authors contributed comments on several manuscript drafts and read and approved the final manuscript.

## Pre-publication history

The pre-publication history for this paper can be accessed here:

http://www.biomedcentral.com/1471-2458/12/628/prepub

## Supplementary Material

Additional file 1 Search strategy conducted in Medline. Click here for file

Additional file 2 Quality appraisal rating procedure. Click here for file

Additional file 3 Table of full-text articles excluded from the systematic review. Click here for file

Additional file 4 Full details of articles included in the systematic review categorized by life course model. Click here for file

Additional file 5 Full quality appraisal of included articles. Click here for file

Additional file 6 Quality appraisal ratings of included articles. Click here for file
