# Supplementary material for: Life course socio-economic position and quality of life in adulthood: a systematic review of life course models
Source: BMC Public Health. 2012 Aug 9;12:628. doi: 10.1186/1471-2458-12-628 (PMC3490823; doi:10.1186/1471-2458-12-628)
Supplement: Additional file 1 — Search strategy conducted in Medline. [file 1471-2458-12-628-S1.doc]

**Additional File 1**

**Search Strategy executed in Ovid MEDLINE(R) In-Process & Other Non-Indexed Citations and Ovid MEDLINE(R)) 2^nd^ January 2012**

1 ((((soci* class* or soci* status* or soci* position* or soci* standing* or social group* or socio?economic group* or occupational group* or occupational class* or occupational status* or occupational position* or occupational standing* or prestige or class* schem* or soci* circumstance* or soci* condition* or occupation* or job* or employment* or career* or NS?SEC or E?SEC or registrar general* or Cambridge scale or CASMIN or CAMSIS or SIOPS or ISEI or EGP) not endogenous glucose production) or Erikson* or Goldthorpe* or treiman* or wright* class* or manual class* or non?manual class*) adj3 (life?course* or life?span* or life?time* or life?cycle* or longitudinal or chang* or mov* or mobil* or climb* or fall* or downward* or upward* or increas* or decreas* or migrat* or pathway* or cumulat* or accumulat* or trajector* or pattern* or time)).mp. (20617)

2 (occupation* mobil* or social* mobil* or intra?generation* mobil* or inter?generation* mobil* or socio?cult* mobil* or class mobility or status mobility or career mobility or job mobility or occupation* attainment or status attainment or class attainment).mp. (10216)

3 (social selection or social causation or health selection or social drift* or health constrain* or accumulat* disadvantage* or cumulat* disadvantage* or cumulat* advantage* or accumulation hypothesis or accumulation model or class trajector* or status trajector* or trajectory model or latent model or pathway model or critical period).mp. (6053)

4 exp career mobility/ (8791)

5 exp social mobility/ (753)

6 exp social class/ (28607)

7 exp social hierarchy/ (1533)

8 (life?course* or life?span* or life?time* or life?cycle* or longitudinal or chang* or mov* or mobil* or climb* or fall* or downward* or upward* or increas* or decreas* or migrat* or pathway* or cumulat* or accumulat* or trajector* or pattern* or time).mp. (7700420)

9 6 or 7 (29995)

10 8 and 9 (15067)

11 1 or 2 or 3 or 4 or 5 or 10 (40885)

12 (CASP?19 or CASP?12 or QoL or HRQoL or WEMWBS or SWEMWBS or SF?12 or SF?36 SF?8 or WHOQOL or OPQOL or ICECAP or WHO?5 or PANAS or SWLS or SHS or Fordyce or bradburn or ryffs or GHQ or EURO?D or CES?D or PWI or ABS).mp. (32594)

13 Quality of life/ (97401)

14 personal satisfaction/ (8970)

15 affect/ (22192)

16 happiness/ (2213)

17 exp mental health/ (18125)

18 (Quality of life or life satisfaction or satisfaction with life or personal satisfaction or life evaluat* or Happ* or Well?being or Mental* health* or Psychological* health* or positive affect or negative affect or affect balance or feeling* or Mood or needs satisfaction or psychological needs).mp. (350494)

19 12 or 13 or 14 or 15 or 16 or 17 or 18 (373559)

20 11 and 19 (2935)

21 limit 20 to english language (2702)
