# Supplementary material for: Life course socio-economic position and quality of life in adulthood: a systematic review of life course models
Source: BMC Public Health. 2012 Aug 9;12:628. doi: 10.1186/1471-2458-12-628 (PMC3490823; doi:10.1186/1471-2458-12-628)
Supplement: Additional file 2 — Quality appraisal rating procedure. [file 1471-2458-12-628-S2.doc]

**Additional File 2**

**Quality appraisal rating procedure**

Response rates:

Over 80% = low risk of bias (+)

60 to 80% = moderate risk of bias (0)

Below 60% = greater risk of bias (-)

Attrition rates:

Below 20% = low risk of bias (+)

20 to 40% = moderate risk of bias (0)

Over 40% = greater risk of bias (-)

Measurement of SEP:

Administrative data or prospective collection = higher quality (+)

Mixed collection methods = average quality (0)

Retrospective collection = lower quality (-)

Sample size:

Over 1000 participants = higher quality (+)

500 to 1000 participants = average quality (0)

Below 500 participants = lower quality (-)

- + equals 3 points; 0 equals 2 points; - equals 1 point
- For response and attrition rates take the lowest score of the two
- If any response or attrition rate not provided, item with lowest score used
- If no response or attrition rate provided score as -
- If method for measuring SEP not specified score as -
- Sum the scores to give an overall rating
- Ratings 3 to 4 = poorer quality; 5 to 7 = average quality; 8 to 9 = higher quality
