# Supplementary material for: Life course socio-economic position and quality of life in adulthood: a systematic review of life course models
Source: BMC Public Health. 2012 Aug 9;12:628. doi: 10.1186/1471-2458-12-628 (PMC3490823; doi:10.1186/1471-2458-12-628)
Supplement: Additional file 3 — Table of full-text articles excluded from the systematic review. [file 1471-2458-12-628-S3.doc]

Additional File 3

Table of full-text articles excluded from the systematic review

| **Reference** | **Reason for exclusion**^a^ |
| --- | --- |
| 1. Argyle, M. (1994). *The psychology of social class*. London, England: Routledge. 2. Bailey, L.L., & Hansson, R.O. (1995). Psychological obstacles to job or career change in late-life. *Journals of Gerontology Series B-Psychological Sciences and Social Sciences,* 50, P280-P288. 3. Bergman, L.R., & Daukantaite, D. (2006). The importance of social circumstances for Swedish women's subjective wellbeing. *International Journal of Social Welfare,* 15, 27-36. 4. Bien, B. (1991). A socio-medical survey of the old citizens of Bialystok, Poland: 5 years longitudinal observation. *Journal of Cross-Cultural Gerontology,* 6, 101-108. 5. Binder, M., & Coad, A. (2010). An examination of the dynamics of well-being and life events using vector autoregressions. *Journal of Economic Behavior & Organization,* 76, 352-371. 6. Birtchnell, J. (1971). Social class, parental social class, and social mobility in psychiatric patients and general population controls. *International psychiatry clinics,* 8, 77-103. 7. Blane, D., Berney, L., Smith, G.D., Gunnell, D.J., & Holland, P. (1999). Reconstructing the life course: health during early old age in a follow-up study based on the Boyd Orr cohort. *Public Health,* 113, 117-124. 8. Blau, P.M. (1956). Social mobility and interpersonal relations. *American sociological review,* 21, 290-295. 9. Breeze, E., Jones, D.A., Wilkinson, P., Latif, A.M., Bulpitt, C.J., & Fletcher, A.E. (2004). Association of quality of life in old age in Britain with socioeconomic position: Baseline data from a randomised controlled trial. *Journal of Epidemiology and Community Health,* 58, 667-673. 10. Brody, C.J., & McRae, J.A., Jr. (1987). Models for Estimating Effects of Origin, Destination, and Mobility. *Social Forces,* 66, 208-225. 11. Brunson, B.H. (1997). Life Satisfaction and Retirement: Military Mid-Life Career Change. *Dissertation Abstracts International, A: The Humanities and Social Sciences,* 57, 3266-a. 12. Budowski, M., Masia, M., & Tillmann, R. (2009). Psychological Health: An Analysis of the Intersection of Cumulative Disadvantage and Partnership Events. *Schweizerische Zeitschrift fur Soziologie/Revue Suisse de sociologie/Swiss Journal of Sociology,* 35, 357-376. 13. Burchardt, T. (2005). Just happiness? Subjective wellbeing and social policy. *Social justice: building a fairer Britain*. London: Politico's. 14. Cairney, J., & Avison, W.R. (1999). Age, Social Structure and Perceived Health. American Sociological Association, 1999. 15. Capsi, A., & Elder, G.H., Jr. (1986). Life satisfaction in old age: linking social psychology and history. *Psychology & Aging,* 1, 18-26. 16. Case, A., & Paxson, C. (2011). The Long Reach of Childhood Health and Circumstance: Evidence from the Whitehall II Study*. *The Economic Journal,* 121, F183-F204. 17. Castro, F.G., Marsiglia, F.F., Kulis, S., & Kellison, J.G. (2010). Lifetime segmented assimilation trajectories and health outcomes in Latino and other community residents.[Erratum appears in Am J Public Health. 2011 Jan;101(1):6]. *American Journal of Public Health,* 100, 669-676. 18. Chan, M., Chen, E., Hibbert, A.S., Wong, J.H.K., & Miller, G.E. (2011). Implicit measures of early-life family conditions: relationships to psychosocial characteristics and cardiovascular disease risk in adulthood. *Health Psychology,* 30, 570-578. 19. Chandola, T., Bartley, M., Sacker, A., Jenkinson, C., & Marmot, M. (2003). Health Selection in the Whitehall II Study, UK. *Social Science & Medicine,* 56, 2059-2072. 20. Chandola, T., Ferrie, J., Sacker, A., & Marmot, M. (2007). Social inequalities in self reported health in early old age: Follow-up of prospective cohort study. *British Medical Journal,* 334, 990-993. 21. Clarke, P.J., Marshall, V.W., Ryff, C.D., & Rosenthal, C.J. (2000). Well being in Canadian seniors: Findings from the Canadian Study of Health and Aging. *Canadian Journal on Aging,* 19, 139-159. 22. Cole, E.R., & Omari, S.R. (2003). Race, class and the dilemmas of upward mobility for African Americans. *Journal of Social Issues,* 59, 785-802. 23. Crosnoe, R., & Elder, G.H., Jr. (2002). Successful Adaptation in the Later Years: A Life Course Approach to Aging. *Social Psychology Quarterly,* 65, 309-328. 24. Dannefer, D., Pallas, A.M., Jennings, J.L., Schafer, M.H., Shippee, T.P., Ferraro, K.F., et al. (2009). Cumulative and compensatory effects over the life course. *Schweizerische Zeitschrift für Soziologie,* 35, 183-404. 25. DuWors, R.E., Jr. (1986). The Consequences of Social Mobility for the Quality of Life and Politics. *Dissertation Abstracts International, A: The Humanities and Social Sciences,* 46, 2446-a. 26. Fleming, N., & Marks, G.N. (1998). Well-Being among Young Australians: Effects of Work and Home Life for Four Youth in Transition Cohorts. *Longitudinal Surveys of Australian Youth*, 1-28. 27. Ford, G., Ecob, R., Hunt, K., Macintyre, S., & West, P. (1994). Patterns of class inequality in health through the lifespan: class gradients at 15, 35 and 55 years in the west of Scotland. *Social Science & Medicine,* 39, 1037-1050. 28. Gilman, S.E. (2002). Childhood socioeconomic status, life course pathways and adult mental health. *International Journal of Epidemiology,* 31, 403-404. 29. Gross, M. (1998). Educational Systems, Intragenerational Mobility and Perceived Social Inequality. International Sociological Association, 1998. 30. Grundy, E., & Sloggett, A. (2003). Health inequalities in the older population: The role of personal capital, social resources and socio-economic circumstances. *Social Science & Medicine,* 56, 935-947. 31. Hans, B., Mheen, H.D.v.d., & Johan, P.M. (1999). Social class in childhood and general health in adulthood: questionnaire study of contribution of psychological attributes. *British Medical Journal,* 318, 18-22. 32. Harrison, J., Barrow, S., & Creed, F. (1998). Mental health in the north west region of England: associations with deprivation. *Social Psychiatry & Psychiatric Epidemiology,* 33, 124-128. 33. Hatch, S.L., Harvey, S.B., & Maughan, B. (2010). A developmental-contextual approach to understanding mental health and well-being in early adulthood. *Social Science &amp; Medicine,* 70, 261-268. 34. Hemingway, H., Nicholson, A., Stafford, M., Roberts, R., & Marmot, M. (1997). The impact of socioeconomic status on health functioning as assessed by the SF-36 questionnaire: the Whitehall II Study. *American Journal of Public Health,* 87, 1484-1490. 35. Hertzman, C. (1999). The biological embedding of early experience and its effects on health in adulthood. *Annals of the New York Academy of Sciences,* 896, 85-95. 36. Huang, Q., El-Khouri, B.M., Johansson, G., Lindroth, S., & Sverke, M. (2007). Women's career patterns: A study of Swedish women born in the 1950s. *Journal of Occupational and Organizational Psychology,* 80, 387-412. 37. Isaksson, K. (1990). A longitudinal study of the relationship between frequent job change and psychological well-being. *Journal of Occupational Psychology,* 63, 297-308. 38. Jacob, M. (2005). Perceived discrimination, cumulative disadvantage and the life course: Women's mental health in retirement/old age. *Gerontologist,* 45, 307-307. 39. Jager, J.O. (2008). The impact of social status on levels on psychological well-being: A dynamic, developmental approach. *Dissertation Abstracts International: Section B: The Sciences and Engineering,* 68, 7001. 40. Kantor, M.B. (1965). Mobility and mental health; proceedings of the Fifth Annual Conference on Community Mental Health Research, Social Science Institute, Washington University 1963. *Springfield,* 247. 41. Kessin, K. (1971). Social and psychological consequences of intergenerational occupational mobility. *American Journal of Sociology,* 77, 1-18. 42. Kim, J., & Durden, E. (2007). Socioeconomic status and age trajectories of health. *Social Science & Medicine,* 65, 2489-2502. 43. Kulis, S. (1987). Socially Mobile Daughters and Sons of the Elderly: Mobility Effects within the Family Revisited. *Journal of Marriage and the Family,* 49, 421-433. 44. Long, J.V., & Vaillant, G.E. (1984). Natural history of male psychological health. XI: Escape from the underclass. *American Journal of Psychiatry,* 141, 341-346. 45. Luo, Y., & Waite, L.J. (2005). The impact of childhood and adult SES on physical, mental, and cognitive well-being in later life. *The journals of gerontology,* Series B, Psychological sciences and social sciences. 60, S93-S101. 46. Lyonette, C., & Yardley, L. (2003). Predicting positive and negative mental health outcomes in working female careers: A longitudinal analysis. *Gerontologist,* 43, 503-503. 47. Marshall, G., & Firth, D. (1999). Social Mobility and Personal Satisfaction: Evidence from Ten Countries. *The British Journal of Sociology,* 50, 28-48. 48. Martin, J.K., & Lichter, D.T. (1983). Geographic Mobility and Satisfaction with Life and Work. *Social Science Quarterly,* 64, 524-535. 49. Matsuura, M., Sugawara, M., & Atushi, S. (2008). Career pattern and mental health of women in the child-rearing years. *International Journal of Psychology,* 43, 287-287. 50. Maurer, J.G. (1969). The downward-mobile industrial supervisor: characteristics and attitudes. *Sociology and Social Research,* 53, 311-322. 51. McDonough, P., & Berglund, P. (2003). Histories of poverty and self-rated health trajectories. *Journal of Health and Social Behavior,* 44, 198-214. 52. McDonough, P., Sacker, A., & Wiggins, R.D. (2005). Time on my side? Life course trajectories of poverty and health. *Social Science & Medicine,* 61, 1795-1808. 53. McIlvane, J.M., Ajrouch, K.J., & Antonucci, T.C. (2007). Generational Structure and Social Resources in Mid-Life: Influences on Health and Well-Being. *Journal of Social Issues,* 63, 759-773. 54. Mensah, F., & Hobcraft, J. (2008). Childhood deprivation, health and development: Associations with adult health in the 1958 and 1970 British prospective birth cohort studies. *Journal of Epidemiology and Community Health,* 62, 599-606. 55. Mueller, M.M. (2003). Work, Family and Well-Being over the Life Course: Continuities and Discontinuities in the Lives of American Women. *Dissertation Abstracts International, A: The Humanities and Social Sciences,* 63, 4108-a. 56. Price, D. (2006). The poverty of older people in the UK. *Journal of Social Work Practice,* 20, 251-266. 57. Regidor, E., Pascual, C., Martínez, D., Ortega, P., Astasio, P., & Calle, M.E. (2011). Heterogeneity in the association between socioeconomic position in early life and adult self-rated health in two birth cohorts of Spanish adults. *Journal of Epidemiology and Community Health,* 65, 999-1005. 58. Roborgh, P., & Stacey, B.G. (1987). Happiness and Radical Career Change among New Zealanders. *The Journal of Psychology,* 121, 501-514. 59. Rose, G., Bengtsson, C., Dimberg, L., Kumlin, L., & Eriksson, B. (1998). Life events, mood, mental strain and cardiovascular risk factors in Swedish middle-aged men. Data from the Swedish part of the Renault/Volvo Coeur Study. *Occupational Medicine,* 48, 329-336. 60. Ryff, C., & Singer, B. (2008). Know Thyself and Become What You Are: A Eudaimonic Approach to Psychological Well-Being. *Journal of Happiness Studies,* 9, 13-39. 61. Schoon, I., Sacker, A., & Bartley, M. (2003). Socio-Economic Adversity and Psychosocial Adjustment: A Developmental-Contextual Perspective. *Social Science & Medicine,* 57, 1001-1015. 62. Siegel, M.J., Akincigil, A., Amin, S., & Crystal, S. (2009). Cumulative advantage, educational attainment, and late life health status. *Schweizerische Zeitschrift für Soziologie,* 35, 377-404. 63. Stansfeld, S.A., Head, J., & Marmot, M.G. (1998). Explaining social class differences in depression and well-being. *Social Psychiatry & Psychiatric Epidemiology,* 33, 1-9. 64. Tiffin, P.A., Pearce, M.S., & Parker, L. (2005). Social mobility over the lifecourse and self reported mental health at age 50: Prospective cohort study. *Journal of Epidemiology and Community Health,* 59, 870-872. 65. Timms, D.W. (1996). Social mobility and mental health in a Swedish cohort. *Social Psychiatry & Psychiatric Epidemiology,* 31, 38-48. 66. Wiggins, R.D., Erzberger, C., Hyde, M., Higgs, P., & Blane, D. (2007). Optimal Matching Analysis Using Ideal Types to Describe the Lifecourse: An Illustration of How Histories of Work, Partnerships and Housing Relate to Quality of Life in Early Old Age. *International Journal of Social Research Methodology,* 10, 259-278. 67. Zhang, X. (2008). Rethinking the social consequences of occupational mobility and status discrepancy: New approaches and data. *Dissertation Abstracts International, A: The Humanities and Social Sciences,* 69, 0394. 68. Zimmermann, E., Stuckelberger, A., & Meyer, P.C. (2006). Effects of Cumulative Disadvantage and Disruptive Life Events on the Physical and Mental Health of Individuals between the Ages of 50-74 Years: Analysis from the Swiss Household Panel (SHP). *Swiss Journal of Sociology,* 32, 527-555.   ^a^ Some studies were excluded for multiple reasons, only one reason is provided here | Not published in scholarly journal  Irrelevant independent variables  Irrelevant independent variables  Irrelevant outcome  Irrelevant outcome  Irrelevant outcome  Irrelevant outcome  Review article  Irrelevant independent variables  Age not specified/under 25 years  Not published in scholarly journal  Irrelevant independent variables  Not published in scholarly journal  Irrelevant independent variables  Irrelevant independent variables  Irrelevant outcome  Irrelevant independent variables  Irrelevant independent variables  Irrelevant independent variables  Irrelevant outcome  Irrelevant independent variables  Review article  Irrelevant independent variables  Review article  Not published in scholarly journal  Irrelevant independent variables  Irrelevant independent variables  Irrelevant outcome  Irrelevant outcome  Irrelevant outcome  Irrelevant outcome  Irrelevant independent variables  Irrelevant outcome  Irrelevant independent variables  Review article  Job, employment status or income mobility  Job, employment status or income mobility  Irrelevant independent variables  Not published in scholarly journal  Not published in scholarly journal  Irrelevant outcome  Irrelevant independent variables  Irrelevant independent variables  Irrelevant independent variables  Irrelevant outcome  Irrelevant independent variables  Age not specified/under 25 years  Irrelevant independent variables  Irrelevant independent variables  Irrelevant outcome  Irrelevant independent variables  Irrelevant independent variables  Irrelevant independent variables  Irrelevant outcome  Not published in scholarly journal  Review article  Irrelevant outcome  Irrelevant independent variables  Irrelevant outcome  Irrelevant independent variables  Irrelevant outcome  Irrelevant outcome  Irrelevant independent variables  Irrelevant outcome  Irrelevant outcome  Irrelevant independent variables  Not published in scholarly journal  Irrelevant outcome |
