# Supplementary material for: Life course socio-economic position and quality of life in adulthood: a systematic review of life course models
Source: BMC Public Health. 2012 Aug 9;12:628. doi: 10.1186/1471-2458-12-628 (PMC3490823; doi:10.1186/1471-2458-12-628)
Supplement: Additional file 4 — Full details of articles included in the systematic review categorized by life course model. [file 1471-2458-12-628-S4.doc]

Additional File 4

Full details of articles included in the systematic review categorised by life course model

| **Reference, number**  **Study name**  **Country** | **Study design**  **Sample size** | **Time period of study** | **Sample description** | **Measures of SEP** | **Life course model**  **Implementation of model** | **Outcomes**  **Age outcome measured** | **Summary of results** |
| --- | --- | --- | --- | --- | --- | --- | --- |
| **Accumulation** |  |  |  |  |  |  |  |
| Mäkinen *et al* (2006), [30]  Helsinki Health Study  Finland | Repeat cross-sectional  N=8970 | 2000, 2001, 2002 | 20% male  Aged 40, 45, 50, 55 or 60 years during survey year | Childhood SEP: parent’s education using low education (primary school or less) or high education (secondary school or vocational training, matriculation or university degree). Highest level of mother/father selected. Other childhood circumstances: bullied, chronic disease, parental divorce, death, mental problem, drinking problem or economic difficulties, (classified into none or 1 or more).  Adulthood SEP: own education level classified as above. | Accumulation  Looked at interaction between childhood SEP & adulthood SEP. | SF-36 MCS. Prevalence of limited functioning (lowest quartile).  Aged 40, 45, 50, 55 or 60 years | Women: low childhood SEP & low adulthood SEP prevalence was 28% (CI: 26, 30). Low childhood SEP & high adulthood SEP 30% (CI: 29, 31). High childhood SEP & low adulthood SEP 22% (CI: 21, 23). High childhood SEP & high adulthood SEP 22% (CI: 21, 23).  Men: low childhood SEP & low adulthood SEP prevalence was 25% (CI: 22, 28). Low childhood SEP & high adulthood SEP 28% (CI: 25, 31). High childhood SEP & low adulthood SEP 17% (CI: 14, 20). High childhood SEP & high adulthood SEP 20% (CI: 18, 22). |
| Otero-Rodríguez *et al* (2010), [37]  Spanish population survey  Spain | Cohort  N=2117 | Wave 1:  2001  Wave 2:  2003 | 45% male  Recruited at age 60+ years | Childhood SEP: father’s occupation classified as social class I (professional, managers, proprietors, clerical)  II (self-employed farm workers), III (skilled and unskilled manual workers), IV (paid farm workers). Classes I and II grouped into high social class, classes III and IV grouped into low social class.  Own education level: low (no education) or high (primary or higher).  Adulthood SEP: current or last occupation of household head (classified as above). | Accumulation  Index summing number of low (adverse) SEP positions (range 0-3). Those with no adverse SEPs used as reference. | Change in SF-36 MCS between wave 1 and 2. Grouped into decline (decrease of >5 points) no change, improvement (increase of >5 points).  60+ years | Risk of decline in MCS highest in those with 3 adverse SEPs OR 2.07 (CI: 1.45, 2.97). OR with 1 adverse SEP=1.36 (CI: 1.00, 1.85). Linear trend p<0.001. OR for improvement in MCS highest with 3 adverse SEPs 1.64 (CI: 1.08, 2.48). OR with 1 adverse SEP=1.47 (CI: 1.03, 2.08). Linear trend p=0.056. |
| Singh-Manoux *et al* (2004), [6]  Whitehall II study  United Kingdom | Cohort  N=6128 | Wave 1: 1985-88  Wave 5: 1997-99  Wave 6: 2000-01 | 72% male  Recruited at age 35-55 years | Childhood SEP: PCA used to divide scale into tertiles based on father’s occupation (Registrar General’s social class scheme) and childhood socioeconomic circumstances (4 item scale: not car owner, financial difficulties, no inside toilet, father/mother unemployed when desired work, four yes answers indicated poor SEP).  Own education level: high (degree or higher degree), intermediate (higher secondary), low (lower secondary or none).  Adulthood SEP: employment grade on entry to study: high (administrative), intermediate (professional and executive), & low (clerical & office support staff). | Accumulation  Constructed trajectories using the 3 SEP indicators, resulting in 27 trajectories. 000= high SEP at 3 time points (SS 0, used as reference), 111= intermediate SEP at 3 time points (SS 3), 222= low SEP at 3 time points (SS 6). | SF-36 MCS. Poor functioning (worst quintile).  50+ years | Women: adverse SEP at 3 time points OR 2.42 (CI: 1.3, 4.4, p<0.05), no linear trend (p=0.14). Highest OR in SS 3, OR 2.77 (CI: 1.5, 4.9). Lowest OR in SS 4, OR 1.52 (CI: 0.8, 2.7).  Men: adverse SEP at 3 time points OR 2.60 (CI: 1.4, 4.9, p<0.05). Linear trend (p<0.0001). Lowest OR in SS 3, OR 1.95 (CI: 1.4, 2.7). Highest OR evident in trajectories 100, 200, and 210. |
| **Latent or pathway** | | | | | | | |
| Huurre *et al* (2003), [38]  Finnish school survey  Finland | Cohort  N=1592 | Wave 1:  1983  Wave 2:  1989  Wave 3:  1999 | 45% male  Recruited at age 16 years | Childhood SEP: father's occupation, categorised into manual/non-manual (based on Central Statistical Office 1975 Standard Classification of Occupations). Mother's occupation used if father’s occupation missing, or education level used if both missing.  Adulthood SEP: own occupation at age 32 classified as above. | Latent model  Looked at wellbeing by parental SEP & adjusted for adulthood SEP to see if any effect remained. | Wellbeing: assessed using self-esteem measure (7 statements e.g. satisfaction with self) measured on 5 point scale. Higher scores indicated lower wellbeing.  32 years | Women: mean wellbeing for respondents with non-manual parents 14.3, manual 15.6 (p=0.001). Adjusting for adulthood SEP (p=0.02).  Men: No difference in wellbeing between respondents with non-manual (13.0) & manual parents (13.5). Adjusted/unadjusted p>0.05. |
| Marmot *et al* (1998), [35]  MIDUS  United States | Cross-sectional  N=3032 | 1995 | 48% male  Aged 25 to 74 years | Childhood SEP: father & mother’s education (BA/graduate degree, some college, high school graduate, less than high school graduate).  Adulthood SEP: own education level classified as above. | Latent model  Tested association between own education & wellbeing, controlling for parent's education, age & race. BA/graduate degree used as reference category. | Wellbeing: six dimensions of positive psychological functioning (e.g. purpose in life, self-acceptance). Lowest quintile=least favourable category.  25 to 74 years | Women: adjusting for adulthood SEP, those with mothers who had no junior high education OR 2.22 (CI: 1.1, 4.7). Those with fathers who had no junior high education OR 0.84 (CI: 0.4, 1.6).  Men: adjusting for adulthood SEP, those with mothers who had no junior high education OR 0.70 (CI: 0.3, 1.6). Those with fathers who had no junior high education OR 1.16 (CI: 0.6, 2.2).  All associations attenuated when adulthood SEP controlled for. |
| Otero-Rodríguez *et al* (2010), [37]  Spanish population survey  Spain | Cohort  N=2117 | Wave 1:  2001  Wave 2:  2003 | 45% male  Recruited at age 60+ years | Childhood SEP: father’s occupation classified as social class I (professional, managers, proprietors, clerical)  II (self-employed farm workers), III (skilled and unskilled manual workers), IV (paid farm workers). Classes I and II grouped into high social class, classes III and IV grouped into low social class.  Own education level: low (no education) or high (primary or higher).  Adulthood SEP: current or last occupation of household head (classified as above). | Latent model  Tested 3 SEP indicators (childhood SEP, education, adulthood SEP) together to see if any had independent effect. High SEP used as reference. | Change in SF-36 MCS between wave 1 and 2. Grouped into decline (decrease of >5 points) no change, improvement (increase of >5 points).  60+ years | Low childhood SEP associated with greatest risk in decline in MCS OR 1.41 (CI: 1.12, 1.77) & improvement OR 1.32 (CI: 1.02, 1.71). Low education associated with risk in decline OR 1.36 (CI: 1.09, 1.71), but not improvement OR 1.29 (CI: 0.99, 1.67). Adult SEP not associated with change, OR for decline 0.98 (CI: 0.78, 1.23) and improvement OR 0.88 (CI: 0.67, 1.14). |
| Laaksonen *et al* (2007) , [29]  Helsinki Health Study  Finland | Repeated cross-sectional  N=8970 | 2000, 2001, 2002 | 20% male  Aged 40, 45, 50, 55 or 60 years during survey year | Childhood SEP: mother & father's education level (part of primary school, primary school, secondary school or vocational training, matriculation/college examination, university degree) & childhood economic difficulties (yes/no).  Adulthood SEP: own education level classified as above. Individual income. Occupational class (manual, routine non-manual, semi-professionals, professionals, managers) derived using the occupational classification of Statistics Finland and City of Helsinki. | Latent & pathway model  Used SEM to model the direct & indirect effects of childhood SEP on HRQoL using latent variables for childhood & adulthood SEP. | SF-36 MCS means  Aged 40, 45, 50, 55 or 60 years | Childhood SEP not directly associated with MCS in men (direct effects -0.02, CI: -0.08, 0.04) or women (0.02, CI: -0.01, 0.05). Increased adulthood SEP associated with poorer MCS in women (direct effects -0.14, CI: -0.17, -0.12) & men (-0.10 CI: -0.15, -0.05). Effect via adulthood SEP women (-0.08), men (-0.06). |
| Mäkinen *et al* (2006), [30]  Helsinki Health Study  Finland | Repeat cross-sectional  N=8970 | 2000, 2001, 2002 | 20% male  Aged 40, 45, 50, 55 or 60 years during survey year | Childhood SEP: parent’s education using low education (primary school or less) or high education (secondary school or vocational training, matriculation or university degree). Highest level of mother/father selected.  Adulthood SEP: own education level classified as above. | Latent & pathway model    Tested association between childhood SEP & MCS scores, controlling for adulthood SEP & adverse childhood circumstances. High education used as reference. | SF-36 MCS. OR for low functioning (lowest quartile).  Aged 40, 45, 50, 55 or 60 years | Women: intermediate childhood SEP OR 0.79 (CI: 0.67, 0.93), low childhood SEP OR 0.76 (CI: 0.65-0.89).  Men: intermediate childhood SEP OR 0.75 (CI: 0.54, 1.03), low childhood SEP OR 0.74 (CI: 0.56, 1.00). |
| **Social Mobility** |  |  |  |  |  |  |  |
| Blane *et al* (2004), [36]  Boyd Orr Cohort  United Kingdom | Cohort  N=254 | Wave 1:  1937-39  Wave 2:  1997-98  Wave 3: 2000 | 47% male  Recruited at age 5 to 14 years | Inter-generational mobility: father’s occupation & respondent’s longest held occupation (manual/non-manual).  Intra-generational mobility: respondent’s occupation (manual/non-manual) aged 25 & 50 years. | Social mobility (inter- & intra-generational)  Up (manual to non-manual), same, down (non-manual to manual). | Mean CASP-19 scores  55+ years | No difference in mean CASP-19 scores between upwardly mobile (inter=42.1, intra=41.8) or downwardly mobile (inter=43.2, intra=41.5) or in same position (inter=41.2, intra=41.5). Inter-generational mobility p=0.51, intra-generational mobility p=0.96. |
| Otero-Rodríguez *et al* (2010), [37]  Spanish population survey  Spain | Cohort  N=2117 | Wave 1:  2001  Wave 2:  2003 | 45% male  Recruited at age 60+ years | Inter-generational mobility: father’s occupation & current or last occupation of household head. Classified as social class I (professional, managers, proprietors, clerical), II (self-employed farm workers), III (skilled and unskilled manual workers), IV (paid farm workers). Classes I & II grouped into high social class, classes III and IV grouped into low social class. | Social mobility (inter-generational)  Created variable combining social class of father & current social class & tested interaction. Those in high social class at both times used as reference. | Change in SF-36 MCS between wave 1 & 2. Grouped into decline (decrease of >5 points) no change, improvement (increase of >5 points).  60+ years | High to low SEP: no association OR 1.07 (CI: 0.76, 1.49). Low to high SEP: more likely to have decline in MCS OR 1.58 (CI: 1.12, 2.22) & improvement OR 1.65 (CI: 1.11, 2.44). Low SEP at both times: greater risk of decline in MCS OR 1.45 (CI: 1.10, 1.90), but not improvement OR 1.20 (CI: 0.87, 1.64). |
| Runyan (1980), [34]  Oakland Growth Study  United States | Cohort  N=91 | Wave 1: 1932  Wave 2:  1958-59 | 49% male  Recruited in grades 5 & 6 of high school. | Inter-generational mobility: father’s occupation & respondent’s occupation aged around 38 years, classified using Hollingshead class scheme (groups 1, 2, 3 =middle class & 4, 5=working class). | Social mobility (inter-generational)  Upward mobility (working to middle class), non-mobile, downward mobility (middle to working class). | Life satisfaction (rated 1-9, 9=high) retrospectively assessed for the past 4 years using life satisfaction chart.  34 to 38 years | Men: downwardly mobile had lowest satisfaction (mean 6.07).  Women: stable group had lowest satisfaction (5.65).  Differences between upward, stable & downward groups not significant. |
| Breeze *et al* (2001), [33]  Whitehall Study  United Kingdom | Cohort  N=7041 | Wave 1:  1967-70  Wave 2:  1997-98 | 100% male  Recruited at age 40 to 69 years | Intra-generational mobility: civil service employment grade at baseline wave & employment grade at retirement from wave 2. Classified as high (senior managers & administrators), middle (executives & professionals in less senior professions), low (clerical, catering staff etc.). | Social mobility (intra-generational)  Upward mobility (higher employment grade category at retirement) compared with same or lower grade. | SF-36 MCS. Poor score defined as below 60% of the maximum.  67-97 years | Those with higher grade at retirement than baseline less likely to have a poor MCS. Middle grade OR 0.82 (CI: 0.6, 1.0), low grade OR 0.44 (CI: 0.3, 0.8). P for interaction=0.033. |
| Houle (2011), [39]  Wisconsin Longitudinal Study  United States | Cohort  N=4992 | Wave 1: 1957  Wave 2:  1975  Wave 3:  1992-93 | 100% male  Around 18 years | Intra-generational mobility:  occupation of respondent aged around 36 years & 52 years classified using 6 class version of EGP scheme, class I (service), II (routine non-manual), III (petty bourgeoisie), IV (farm), V (skilled manual), VI (unskilled manual). | Social mobility (intra-generational)  Created dummy variables for downwardly mobile (out of class I or III into II, IV, V or VI), or as result of involuntary job loss, upwardly mobile (from class II, IV, V or VI into class I or III) & all other forms of intra-generational mobility (horizontal, voluntary downward). Non-mobile=reference.  Used diagonal mobility models to model mobility effects. | Wellbeing: self-acceptance subscale of Ryff's psychological wellbeing scale.  Around 52 years | Mobility not associated with wellbeing. Coefficients for downward mobility (B=-0.112, SE=0.130), upward mobility (B=0.042, SE=0.093) & other mobility (B=0.029, SE=0.041), p>0.10. Mobile individuals more likely to report levels of wellbeing that resemble their current class (B=0.657, SE=0.114, p≤0.001) than their prior class (B=0.343, SE=0.114, p≤0.01). |
| Huang and Sverke (2007), [31]  Individual Development and Adaptation Cohort  Sweden | Cohort  N=291 | Wave 1:  1965  Wave 2:  1998 | 100% female  Recruited at age 10 years | Intra-generational mobility: respondent’s occupational history from ages 16 to 43 years using Swedish Standard Classification of Occupations 1996. | Social mobility (intra-generational)  Used optimal matching and cluster analysis to identify career patterns (upward mobility, stable, downward mobility). | Life satisfaction (rated 1-8, 8=high)  43 years | No difference in mean life satisfaction between upward/stable/downward mobility patterns F=1.32 (p>0.05). |
| Johansson *et al* (2007), [32]  Individual Development and Adaptation Cohort  Sweden | Cohort  N=514 | Wave 1:  1965  Wave 2:  1998  Wave 3:  2004 | 100% female  Recruited at age 10 years | Intra-generational mobility: respondent’s occupational history from ages 16 to 43 years using Swedish Standard Classification of Occupations 1996. | Social mobility (intra-generational)  Used optimal matching and cluster analysis to identify career patterns (upward mobility, stable, downward mobility). | Life satisfaction: SWLS (rated 1-7, 7=high)  Wellbeing: Ryff's psychological well-being scale  49 years | No difference in mean life satisfaction between upward/stable/downward mobility patterns F=1.75 (p>0.05).  Mean wellbeing in upwardly mobile 85.00 (entrepreneurs), 83.20 (professionals), 84.30 (associate professionals), compared with 74.56 in downwardly mobile (unskilled workers) F=3.17 (p=<0.001). |

B= beta coefficient; BCS70=1970 British Cohort Study; CASP-19= control, autonomy, self-realisation & pleasure; CI= 95% confidence interval; EGP: Erikson-Goldthorpe-Portocarero class scheme; HRQoL= health-related quality of life; MCS= mental component summary; MIDUS= National Survey of Mid-Life Development in the United States; NCDS=National Child Development Survey 1958; OR= odds ratio; PCS: principal component analysis; SE= standard error; SEM= structural equation modelling; SEP= socio-economic position; SF-36= short-form 36; SWLS= The Satisfaction with Life Scale
