# Supplementary material for: Life course socio-economic position and quality of life in adulthood: a systematic review of life course models
Source: BMC Public Health. 2012 Aug 9;12:628. doi: 10.1186/1471-2458-12-628 (PMC3490823; doi:10.1186/1471-2458-12-628)
Supplement: Additional file 5 — Full quality appraisal of included articles. [file 1471-2458-12-628-S5.doc]

Additional File 5

Full quality appraisal of included articles

| **Reference**  **Number** | **Study design** | **Sampling procedure**  **Representativeness** | **Final sample size** | 1. **Attrition rate** 2. **Attrition reasons specified** 3. **Response rates** | 1. **Did those lost to attrition differ?** 2. **Did non-responders differ?** | **Measurement of SEP variables** | **Missing data reported** | **Analytical strategy for dealing with missing data** | **Variables controlled for** |
| --- | --- | --- | --- | --- | --- | --- | --- | --- | --- |
| Blane *et al* (2004)  [36] | Cohort | Wave 1: Non-random sample of 16 survey centres. Wave 2: stratified random sample.  Considered representative of British population of similar age. | 254 | 1. Wave 3: 4% 2. Yes (death, illness, untraceable) 3. From references: Wave 1: Over two thirds. Wave 2: 43%. Wave 3: 90% of those who responded in wave 2. | 1. NS 2. Non-responders not different in social class or gender but more likely to have limiting long-term illness. | Father’s occupation measured prospectively. Respondent’s occupational history recorded via retrospective recall using life-grid method. | No | Complete case analysis for each item | No variables adjusted for in inter-generational or intra-generational mobility analyses. Age controlled for in other analyses within the publication |
| Breeze *et al* (2001)  [33] | Cohort | All Whitehall Civil Servants aged around 40-69 years  Considered representative of Civil Service employees of that age group | 7041 | 1. Overall: 55% 2. Yes (death or migration abroad) 3. Wave 2: 82% | 1. NS 2. Non-responders more likely to be in low employment grade, older, smokers & have increasing symptoms of cough/phlegm at baseline | Prospectively | Approximately 4% were missing MCS score | Complete case analysis for each item | Age, gender, marital status, smoking |
| Houle (2011)  [39] | Cohort | Random sample of high school graduates in Wisconsin during 1957.  Considered representative of non-Hispanic white American high school graduates in late 1950s. | 4992 | From references:   1. Overall: 6% 2. Yes (death) 3. Original NS. 1975 wave: 89%. 1992-93 wave: 80%. | 1. NS 2. NS | Prospectively | No | Multiple imputation | Age, gender, depression history, marital status in 1975, cognitive ability (human capital), educational attainment in 1975, years in current social class, limiting health conditions, employment status in 1992, recent stressful life event |
| Huang and Sverke (2007)  [31] | Cohort | All children in grades 3, 6 & 8 in a mid-size Swedish urban community during 1960s.  Considered reasonably representative of overall Swedish female population. | 291 | 1. Wave 2: 6%. Wave 3: 8% from baseline. 2. NS 3. Wave 2: 89%. Wave 3: 82%. | 1. NS 2. NS | Retrospective recall via life plot | 20 lacked complete occupational histories. 258 missing life satisfaction data | Complete case analysis | Age, gender |
| Huurre (2003)  [38] | Cohort | All 9th grade pupils attending secondary school in the spring of 1983 in Tampere, Finland.  NS. | 1592 | 1. Wave 2: 2.5%. Wave 3: 2.2%. 2. Yes (death, incomplete or missing ID numbers, address not found, institutionalisation) 3. Wave 1: 97% Wave 2: 77% Wave 3: 70%. | 1. Non-participants more often male, had poorer school performance, frequently used alcohol & smoked 2. NS | Prospectively | No | Complete case analysis | Age, gender |
| Johansson *et al* (2007)  [32] | Cohort | All children in grades 3,6 & 8 in a mid-size Swedish urban community.  Considered reasonably representative of overall Swedish female population. | 514 | 1. Wave 2: 6%. Wave 3: 8% from baseline. 2. NS 3. Wave 2: 89%. Wave 3: 82%. | 1. NS 2. NS | Retrospective recall via life plot | 258 missing life satisfaction data | Complete case analysis | Age, gender |
| Laaksonen *et al* (2007)  [29] | Repeat cross-sectional | All employees aged 40, 45, 50, 55 or 60 years at time of survey employed by the City of Helsinki, identified from personnel register.  Generally representative of target population, younger people & manual workers slightly underrepresented. | 8970 | 1. N/A 2. N/A 3. Overall 67% | 1. N/A 2. N/A | Retrospective recall, apart from income (derived from employer’s personnel register). | 22% had missing income data | Complete case analysis except cases with missing income data included in analysis | Age, gender, material circumstances |
| Mäkinen *et al* (2006)  [30] | Repeat cross-sectional | All employees aged 40, 45, 50, 55 or 60 years at time of survey employed by the City of Helsinki, identified from personnel register  Generally representative of target population, younger people & manual workers slightly underrepresented | 8970 | 1. N/A 2. N/A 3. Overall 67% | 1. N/A 2. N/A | Retrospective recall | No | Complete case analysis | Age, gender, adverse childhood circumstances |
| Marmot *et al* (1998)  [35] | Cross-sectional | Telephone sample followed by self-completed mail questionnaire.  Representative of non-institutionalised population in the United States aged 25-84 who had a telephone. | 3032 | 1. N/A 2. N/A 3. Telephone interview: 70%. Mail questionnaire: 87%.   Overall: 61%. | 1. N/A 2. NS | Retrospective recall | No | If at least 75% of items were completed in scale the mean was imputed. If fewer than 75% completed the scale was classified as missing & excluded | Age, gender, race |
| Otero-Rodríguez *et al* (2010)  [37] | Cohort | NS  Considered representative of the non-institutionalised Spanish population  aged over 60 years. | 2117 | 1. 19% 2. Yes (229 died) 3. NS | 1. Less likely to report more chronic illness. 2. More likely to be older, lower educated, more frequently sedentary, lower alcohol consumption & abdominal obesity. | Retrospective recall | 263 lacked SEP measure,  626 lacked SF-36 | Complete case analysis | Baseline SF-36, age, sex, marital status, cohabitation, smoking, alcohol consumption, physical activity, obesity, health service use, chronic illnesses |
| Runyan (1980)  [34] | Cohort | Sample of 212 5th and 6th graders in Oakland, California.  NS | 91 | 1. 57% 2. NS 3. NS | 1. NS 2. NS | NS | No | Complete case analysis | Age, gender |
| Singh-Manoux *et al* (2004)  [6] | Cohort | Contacted all  London-based office staff, aged 35–55, working in 20 Civil Service departments.  Considered representative of white-collar Civil Service employees of that age group. | 6128 | 1. 3% from phase I to V 2. Yes (355 died) 3. Phase I: 73% | 1. NS 2. Missing data more common among lower employment grades | Childhood SEP & education via retrospective recall. Adult employment grade measured prospectively. | 3825 of 9953 respondents had missing data. | Complete case analysis | Age, gender |

MCS= mental component summary; N/A= not applicable; NS= not specified; SEP= socio-economic position; SF-36= short form-36
