# Supplementary material for: Life course socio-economic position and quality of life in adulthood: a systematic review of life course models
Source: BMC Public Health. 2012 Aug 9;12:628. doi: 10.1186/1471-2458-12-628 (PMC3490823; doi:10.1186/1471-2458-12-628)
Supplement: Additional file 6 — Quality appraisal ratings of included articles. [file 1471-2458-12-628-S6.doc]

**Additional File 6**

**Quality appraisal ratings of included articles**

| **Reference, number** | **Response & attrition rates** | **Sample size** | **Measurement of SEP** | **Rating** | **Overall quality** |
| --- | --- | --- | --- | --- | --- |
| Blane *et al* 2004, [36] | - | - | 0 | 4 | Poorer |
| Breeze *et al* 2001, [33] | - | + | + | 7 | Average |
| Houle 2011, [39] | + | + | + | 9 | Higher |
| Huang and Sverke 2007, [31] | + | - | - | 5 | Average |
| Huurre 2003, [38] | 0 | + | + | 8 | Higher |
| Johansson *et al* 2007, [32] | + | 0 | - | 6 | Average |
| Laaksonen *et al* 2007, [29] | 0 | + | 0 | 7 | Average |
| Mäkinen *et al* 2006, [30] | 0 | + | - | 6 | Average |
| Marmot *et al* 1998, [35] | 0 | + | - | 6 | Average |
| Otero-Rodríguez *et al* 2010, [37] | + | + | - | 7 | Average |
| Runyan 1980, [34] | - | - | - | 3 | Poorer |
| Singh-Manoux *et al* 2004, [6] | 0 | + | 0 | 7 | Average |

+ equals 3 points; 0 equals 2 points; - equals 1 point

Ratings 3 to 4 = poorer quality; 5 to 7 = average quality; 8 to 9 = higher quality
